# Sexual Risk Behaviours and Willingness to Be Circumcised among Uncircumcised Adult Men in Uganda

**DOI:** 10.1371/journal.pone.0144843

**Published:** 2015-12-14

**Authors:** Simon P. S. Kibira, Fredrick Makumbi, Marguerite Daniel, Lynn Muhimbuura Atuyambe, Ingvild Fossgard Sandøy

**Affiliations:** 1 Centre for International Health, Department of Global Public Health and Primary Care, University of Bergen, Bergen, Norway; 2 Department of Community Health and Behavioural Sciences, Makerere University School of Public Health, Kampala, Uganda; 3 Department of Epidemiology and Biostatistics, Makerere University School of Public Health, Kampala, Uganda; 4 Department of Health Promotion and Development, University of Bergen, Bergen, Norway; International AIDS Vaccine Initiative, UNITED STATES

## Abstract

**Background:**

There has been substantial demand for safe male circumcision (SMC) in Uganda in the early programme scale-up phase. Research indicates that early adopters of new interventions often differ from later adopters in relation to a range of behaviours. However, there is limited knowledge about the risk profile of men who were willing to be circumcised at the time of launching the SMC programme, i.e., potential early adopters, compared to those who were reluctant. The aim of this study was to address this gap to provide indications on whether it is likely that potential early adopters of male circumcision were more in need of this new prevention measure than others.

**Methods:**

Data were from the 2011 Uganda AIDS Indictor Survey (UAIS), with a nationally representative sample of men 15 to 59 years. The analysis was based on generalized linear models, obtaining prevalence risk ratios (PRR) with 95% confidence intervals (CI) as measures of association between willingness to be circumcised and multiple sexual partners, transactional sex, non-marital sex and non-use of condoms at last non-marital sex.

**Results:**

Of the 5,776 men in the survey, 44% expressed willingness to be circumcised. Willingness to be circumcised was higher among the younger, urban and educated men. In the unadjusted analyses, all the sexual risk behaviours were associated with willingness to be circumcised, while in the adjusted analysis, non-marital sex (Adj PRR 1.27; CI: 1.16–1.40) and non-use of condoms at last such sex (Adj PRR 1.18; CI: 1.07–1.29) were associated with higher willingness to be circumcised.

**Conclusion:**

Willingness to be circumcised was relatively high at the launch of the SMC programme and was more common among uncircumcised men reporting sexual risk behaviours. This indicates that the early adopters of SMC were likely to be in particular need of such additional HIV protective measures.

## Introduction

There are several biomedical and behavioural interventions available to reduce the impact of the HIV epidemics in sub Saharan Africa, and partly as a result of this, incidence is declining in most of the region [1]. Voluntary medical male circumcision, also known as safe male circumcision, is one of the most recent such interventions. The foreskin is one of the prime sites for HIV entry [2] and male circumcision reduces heterosexual HIV transmission risk from infected women to men as indicated in several observational studies [3–5] and randomised controlled trials in Uganda [6], Kenya [7], and South Africa [8]. It also reduces the prevalence of high risk human papilloma virus that is most associated with cervical cancer [9] and incidence of herpes simplex virus infection among men [10], and genital ulcers in female partners of circumcised HIV negative men [11]. As a result of the overwhelming beneficial evidence, WHO and UNAIDS in 2007 recommended adoption of safe male circumcision (SMC) in fourteen priority countries with high HIV prevalence and low male circumcision levels, including Uganda [12, 13].

Following the WHO recommendation, the Uganda Ministry of Health has since 2007 implemented the safe male circumcision programme through activities aimed at educating leaders, health workers and the general public about SMC [14–16]. In the first years these efforts included public debates, radio and television talk shows, educational materials for health workers and their clients (flip charts, question and answer booklets for health workers, and brochures for men), and education and counselling through a national health hotline with counsellors [16, 17]. Between 2008 and 2009, over 350 health workers were also trained as trainers for their colleagues [18].

In 2011, the prevalence of male circumcision in Uganda among adult men 15–49 years was 27% [19] and until the WHO recommendation it was mainly practised for cultural and religious reasons among a few ethnic groups. As a result of the implementation of the safe male circumcision intervention, demand and service provision have increased. By September 2013, 1,117 health facilities offered SMC services, and from 2008 to 2013, one million four hundred thousand adult men were circumcised; 800,000 between October 2012 and September 2013 alone [15, 20, 21].

It is likely that those who have expressed willingness to be circumcised after the implementation of the safe male circumcision programme represent ‘early adopters’ [22] of the intervention in the Uganda. According to the diffusion of innovation theory, early adopters tend to have high social status, above-average education and are not particularly focused on traditions [22]. A study in Kenya found that early adopters of male circumcision perceived themselves to be at higher risk than later adopters [23]. Thus it is possible that potential early adopters may have a different sexual risk profile than the later adopters and those that do not get circumcised. However, there are few published studies elsewhere [23–25] and none in Uganda that have assessed the associations between sexual risk behaviours and willingness to be circumcised in the general population. In a country with a severe generalised HIV epidemic [19] and fears of increased rate of new infections [1], examining willingness to be circumcised among uncircumcised men with varied sexual behaviours is important to assess whether the National safe male circumcision programme seems to be reaching those that have the highest need of increased protection. In conceptualising this study, we hypothesised that uncircumcised men who had higher sexual risk behaviours were more likely to be willing to be circumcised than their counterparts. We therefore set out to compare the sexual risk profile of men who were willing to be circumcised to those who were reluctant in the 2011 UAIS.

## Methods

This study is based on data from the 2011 UAIS, which among other objectives obtained information about HIV/AIDS programme coverage indicators, such as sexual behaviour related to HIV. The survey sample was designed to produce representative estimates for the entire country, urban and rural areas separately, and for each region. The sample size considerations included an adult HIV prevalence of 6.4 obtained from the preceding national survey, a 10% relative error, a design effect of 1.69 and a response rate of 92% of adults for HIV testing [19]. A stratified two-stage cluster sampling design was used. Clusters were selected from each stratum at the first stage, while the second stage involved selecting households for interview to obtain eligible respondents. The strata were defined by urban/rural residence and geographical region. The clusters were from a list of enumeration areas obtained in the 2010 Uganda National Household Survey update of the 2002 Uganda Population Census. A total of 470 clusters were selected from the strata at the first stage, while the second stage involved systematically sampling 25 households for interview in each cluster to obtain a self-weighting sample. A total of 11,340 occupied households were interviewed, and in these households 9,588 men completed individual interviews. Eligible respondents were permanent residents of the households or visitors who had spent a night in the household before the survey. This paper is based on 5,776 cases of men age 15–59 years who were uncircumcised and reported to ever have had sex at the time of the survey.

### Data collection and variables

The data were collected between February and September 2011 and the survey was led by the Ministry of Health working with ICF international, USA and Uganda Bureau of Statistics. Individual male interviews obtained data on respondents’ self-reported circumcision status, willingness to be circumcised, their reported sexual behaviours, personal perceived risk of HIV infection, knowledge of the protection offered by male circumcision against HIV infection, and socio-demographic characteristics (age, marital status, highest education level, survey region, ethnicity, residence, religion). Information on wealth status was obtained from the household questionnaire and reflects the state of the household in which a man was interviewed, and not necessarily the wealth level of the individual men.

The primary outcome was willingness to be circumcised among all uncircumcised men in the sample. Men who had not decided whether they would like to be circumcised (3.9%) were recoded as unwilling. Our main independent variables were the following sexual risk behaviours [26]: (i) having multiple sexual partners in the 12 months preceding the survey, (ii) transactional sex (payment or receipt of money/gifts in exchange for sex) in the 12 months preceding the survey (iii) having had sex with a non-marital partner in the 12 months preceding the survey, and (iv) non-use of condoms at the last non-marital sex. ‘Multiple sexual partners’ was defined as reporting two or more sexual partners. Non-marital sex and condom use at last non-marital sex were collapsed into one variable with three levels; did not have non-marital sex in the previous 12 months, did not use a condom at last non-marital sex, used a condom at last non marital sex. This was done to ensure that we had a complete sample of all uncircumcised men for the multivariable model, not only the sub sample that reported non-marital sex. Other explanatory variables were socio-demographic characteristics. In order to have more power to detect difference in willingness between different subgroups, we merged some of the original categories for some of the variables: All men who reported living with a woman as if married were coded as “currently married”, and ethnic groups were categorised into those that are geographically close to each other or who share the tradition of male circumcision although their areas of origin are distant geographically. For example, the Bagisu, Sabiny and the Bakonjo were categorised into one group because they are traditionally circumcising ethnic groups in Uganda even though the Bakonjo are from a different region.

### Statistical analyses

The analyses were conducted using STATA version 13 (StataCorp 2013). We explored variables of interest at univariate level, including examining their distribution and missing data. To estimate the associations between the sexual risk behaviours and willingness to be circumcised, we used a ‘modified’ Poisson regression model via a generalized linear model with family (Poisson) and link (log), obtaining prevalence risk ratios (PRR) with their 95% confidence intervals (CI) as a measure of association. PRRs were used because the outcome variable had prevalence above 10% [27–29]. We checked for correlation between the independent variables. In the multivariable analysis with all the sexual risk behaviours, we also adjusted for potential confounding from socio-demographic variables. Marital status was excluded in the multivariable analysis because it was highly correlated with non-marital sex and condom use at last such sex. Sample weights were used in order to account for differential non-response in the survey and we adjusted for clustering.

### Ethical considerations

The UAIS 2011 was reviewed and approved by the Science and Ethics Committee of the Uganda Virus Research Institute, ICF International’s Institutional Review Board, and a review committee at the Centers for Disease Control and Prevention in Atlanta. It was also cleared by the Ethics Committee of the Uganda National Council of Science and Technology. We obtained permission to use the UAIS data from ICF international, USA and the Uganda Ministry of Health.

## Results

### Description of uncircumcised men

Table 1 presents the characteristics of 5,776 uncircumcised men. Forty four percent (2,516) of uncircumcised men were willing to be circumcised. There was a higher prevalence of willingness to be circumcised among younger men aged 15 to 24 (59.3%) and 25 to 34 years (48.9%), men from urban areas (49.7%) those with secondary (50.6%) or higher education (47.1%) as well as among those from households in the top two wealth quintiles. Forty seven percent of uncircumcised men who perceived themselves to be at high risk of contracting HIV were willing to be circumcised compared to 42.6% of those who had low self-perceived risk (chi square p value = 0.006). Among uncircumcised men who knew that circumcision was protective against HIV, 58.8% were willing to be circumcised while only 31% of those who did not have this knowledge were willing.

**Table 1:** Characteristics of uncircumcised men willing to be circumcised and those who were not willing, Uganda 2011.

Variables	Willingness to be circumcised		
	Not willing (%)	Willing (%)	Total
**Age**			
15–24	546 (40.7)	795 (59.3)	1341 (100)
25–34	903 (51.1)	864 (48.9)	1767 (100)
35–44	925 (61.7)	574 (38.3)	1498 (100)
45–59	886 (75.8)	283 (24.2)	1169 (100)
**Residence**			
Urban	470 (50.3)	465 (49.7)	935 (100)
Rural	2790 (57.6)	2051 (42.4)	4841 (100)
**Highest education level**			
No Education	305 (70.0)	131 (30.0)	436 (100)
Primary	1965 (58.3)	1406 (41.7)	3371 (100)
Secondary	722 (49.4)	740 (50.6)	1462 (100)
Higher	268 (52.9)	239 (47.1)	507 (100)
**Survey region**			
Central	648 (49.9)	651 (50.1)	1299 (100)
Kampala	167 (46.4)	192 (53.6)	359 (100)
Eastern	394 (47.9)	429 (52.2)	823 (100)
Northern	1256 (69.5)	551 (30.5)	1807 (100)
Western	795 (53.5)	692 (46.5)	1488 (100)
**Wealth quintile**			
Lowest	777 (67.3)	377 (32.7)	1154 (100)
Second	699 (61.0)	447 (39.0)	1146 (100)
Middle	604 (55.0)	494 (45.0)	1098 (100)
Fourth	590 (51.8)	550 (48.2)	1140 (100)
Highest	590 (47.7)	647 (52.3)	1237 (100)
**Marital status**			
Never married	475 (41.6)	668 (58.5)	1142 (100)
Married	2540 (60.5)	1657 (39.5)	4197 (100)
Divorced/Separated	246 (56.3)	191 (43.7)	437 (100)
**Ethnicity**			
Baganda	480 (51.6)	451 (48.5)	931 (100)
Banyankore	362 (52.5)	328 (47.5)	689 (100)
Iteso/ Karimajong	433 (64.5)	238 (35.5)	671 (100)
Lugbara/Madi/ Alur/Jopadhola	352 (57.9)	255 (42.1)	607 (100)
Basoga	180 (44.9)	221 (55.1)	400 (100)
Langi/Acholi	641 (72.7)	240 (27.3)	881 (100)
Bakiga/Bafumbira	319 (60.2)	211 (39.8)	530 (100)
Bagisu/Sabiny/ Bakonzo	5 (14.3)	30 (85.7)	35 (100)
Banyoro/Batooro	240 (46.2)	280 (53.8)	520 (100)
Others	250 (48.8)	262 (51.2)	512 (100)
**Religion**			
Catholic	1711 (59.0)	1188 (41.0)	2899 (100)
Anglican	1178 (52.9)	1049 (47.1)	2227 (100)
Pentecostal	227 (63.2)	132 (36.8)	359 (100)
Others	145 (49.5)	147 (50.5)	292 (100)
**Perceived HIV risk**			
Low risk	2189 (57.5)	1621 (42.6)	3810 (100)
High risk/not sure	904 (52.6)	814 (47.4)	1718 (100)
Missing	167 (67.3)	81 (32.7)	248 (100)
**Knows SMC reduces HIV risk**			
No	2103 (69.0)	945 (31.0)	3048 (100)
Yes	1091 (41.2)	1556 (58.8)	2647 (100)
Missing	66 (81.1)	15 (18.9)	81 (100)
**Used a condom at last non-marital sex**	** **	** **	** **
Did not have non marital sex	2628 (62.4)	1586 (37.6)	4214 (100)
Did not use a condom	371 (44.8)	456 (55.2)	827 (100)
Used a condom	262 (35.6)	473 (64.4)	735 (100)
**Had multiple sexual partners**	** **	** **	** **
No	2678 (58.1)	1933 (41.9)	4611 (100)
Yes	582 (49.96)	583 (50.04)	1165 (100)
**Transactional sex**	** **	** **	** **
No	3217 (57.1)	2420 (42.9)	5637 (100)
Yes	43 (30.9)	96 (69.1)	140 (100)
**Total**	**3260 (56.4)**	**2516 (43.6)**	**5776 (100)**

Nearly seven in ten men who had transactional sex in the 12 months preceding the survey were willing to be circumcised compared to only 42% of those who did not report such sex. Among men who reported sex with a non-marital partner and used a condom at the last such sex, 64.4% were willing to be circumcised, while among those who did not use condoms at last non-marital sex, 55.2% were willing. Only 37.2% among those who did not report sex with a non-marital partner were willing to be circumcised (Table 1).

### Association between sexual risk behaviours and willingness to be circumcised

In Table 2, all the sexual risk behaviours were significantly associated with willingness to be circumcised in the unadjusted analyses. Uncircumcised men who reported having multiple sexual partners in the 12 months preceding the survey were more likely to be willing to be circumcised (PRR 1.19; 95% CI: 1.11–1.29) than those who did not report multiple sexual partners. We explored if there were differences among men who reported only two sexual partners and those that had three or more, but this was not significant (results not shown). Men who reported to have engaged in transactional sex in the 12 months period were also significantly more likely to be willing to be circumcised than their counterparts (PRR 1.61; 95% CI: 1.39–1.87). However, the adjusted associations were not significant for these two sexual risk behaviours.

**Table 2:** Generalised linear models showing unadjusted and adjusted associations between willingness to be circumcised and sexual risk behaviours and socio-demographic variables among uncircumcised men age 15–59 years, Uganda 2011.

	Willing to be circumcised. PRR [95% CI]	
	Unadjusted	Multivariable model
**Used a condom at last non-marital sex**		
Did not have non marital sex	1.00	1.00
Did not use a condom	1.47* [1.35,1.59]	1.18* [1.07,1.29]
Used a condom	1.71* [1.59,1.85]	1.27* [1.16,1.40]
**Had multiple sexual partners**		
No	1.00	1.00
Yes	1.19* [1.11,1.29]	1.05 [0.97,1.14]
**Transactional sex**		
No	1.00	1.00
Yes	1.61* [1.39,1.87]	1.14 [0.97,1.33]
**Age**		
15–24	2.45* [2.17,2.77]	2.13* [1.87,2.42]
25–34	2.02* [1.78,2.29]	1.92* [1.69,2.17]
35–44	1.58* [1.38,1.81]	1.53* [1.34,1.75]
45–59	1.00	1.00
**Survey region**		
Northern	1.0	1.00
Central	1.64* [1.49,1.82]	1.48* [1.33,1.65]
Kampala	1.76* [1.55,1.99]	1.45* [1.22,1.72]
Eastern	1.71* [1.55,1.89]	1.61* [1.46,1.79]
Western	1.53* [1.38,1.69]	1.37* [1.23,1.52]
**Residence**		
Rural	1.00	1.00
Urban	1.17* [1.08,1.28]	0.89[0.78,1.01]
**Wealth quintile**		
Lowest	1.00	1.00
Second	1.19* [1.06,1.34]	1.07 [0.96,1.20]
Middle	1.38* [1.23,1.54]	1.14* [1.02,1.28]
Fourth	1.48* [1.32,1.65]	1.18* [1.05,1.32]
Highest	1.60* [1.44,1.79]	1.22* [1.07,1.40]
**Highest Education level**		
No education	1.00	1.00
Primary	1.39* [1.17,1.65]	1.20* [1.02,1.41]
Secondary	1.69* [1.42,2.01]	1.29* [1.09,1.53]
Higher	1.57* [1.28,1.92]	1.27* [1.04,1.55]
**Religion**		
Catholic	1.00	1.00
Anglican	1.15* [1.07,1.23]	1.07 [1.00,1.14]
Pentecostal	0.90 [0.76,1.06]	0.87 [0.75,1.01]
Others	1.23* [1.08,1.41]	1.15* [1.02,1.30]
***Number of men***	***5682***	***5682***

*p<0.*05*

Uncircumcised men who reported use of condoms at the last sex with a non-marital partner in the 12 months period were 1.71 times more likely to be willing to be circumcised than those who did not have non-marital sex, while those who had sex with a non-marital partner without condoms were also 1.47 times more likely to be willing to be circumcised that those who did not report non-marital sex. In the multivariable model the associations were still significant, and those who did used a condom during the last non-marital sexual intercourse appeared to be even more willing than those who did not use a condom (but there was a minor overlap in the confidence intervals for the PRR for the two categories) (Table 2).

Other factors in the adjusted model that were independently associated with willingness to be circumcised were: age, region of residence, wealth quintile of the man’s household, education and religion. Willingness to be circumcised increased with decreasing age. Uncircumcised educated men were more likely to be willing to be circumcised than their uneducated colleagues, while men from households in the middle to highest wealth quintiles were more likely to be willing to be circumcised than those from the lowest wealth quintile. Men from the northern region were the least likely to be willing to be circumcised compared to all other survey regions (Table 2).

## Discussion

This study found high levels of willingness to be circumcised among uncircumcised men who reported sexual risk behaviours than those who did not report such behaviours in the 2011 UAIS. Forty four percent of men were willing to be circumcised. Our results indicate a higher likelihood of willingness to be circumcised among men who reported sex with multiple partners and transactional sex, as well as among those reporting sex with a non-marital partner. In the multivariable model those who did not use a condom during the last non-marital sex in preceding 12 months were most likely to report willingness to be circumcised. These results support the a priori hypothesis. Other factors associated with willingness to be circumcised were; young age, urban residence, higher wealth quintile of the man’s household, having an education, and not being from northern region.

The findings in this study indicate that the willingness to be circumcised was higher among those that had engaged in more risky behaviours. In other recent cross sectional studies conducted in 2010/ 2011 in Zimbabwe [24] and 2008 in Botswana [25], willingness to be circumcised was also associated with more risky sexual behaviours such as having multiple sexual partners [24, 25], non-marital partners, and having engaged in transactional sex [24]. Even those who had used a condom at last non-marital sex were more willing than those who had not had non-marital sex in the preceding year, which could indicate that they did not think condoms gave full protection or that they had not used condoms consistently at all higher risk sexual encounters. Men who engage in sexual risk behaviours may see circumcision as protection from the risk of HIV or other sexually transmitted infections. This could explain their willingness to be circumcised. It could also further indicate that those in most need of further HIV protection are actually the easiest to reach for circumcision. However, such men may need tailored interventions after circumcision to reduce their sexual risk behaviours, and in particular to reduce the probability of sexual risk compensation [30]. Interventions that target continuation or enhancement of consistent use of condoms and reduction in number of sexual partners would probably positively affect behaviour among men who undergo circumcision.

There was a consistently inverse relationship between increasing age group and willingness to be circumcised. Younger uncircumcised men were more likely to be willing to be circumcised, and this was consistent across both the bivariate and multivariable analyses. Circumcision is probably more appealing to younger men compared to older ones because relatively younger men may have a higher personal perception of HIV risk, for which circumcision is protective. Younger men are also more likely to be innovators and early adopters of new interventions [22] such as circumcision. Similar associations between young age and interest in circumcision were found in a Zimbabwe study [24].

Men from the northern region were least likely to be willing to circumcise than all other regions. This region also had the lowest prevalence in the country at the time of the survey in 2011 [19, 31]. It is difficult to find a plausible explanation for the low willingness to be circumcised, although cultural traditions could have played a role [32].

Education was positively associated with willingness to be circumcised. Education plays a positive role in acceptance of health interventions and more educated men may easily seek more information than the uneducated counterparts. Other studies have also found associations between education and willingness to be circumcised or circumcision preference [24, 25, 33]. In exploring the relationship between variables in the data, we found educated men to have a higher knowledge about the protective effect of circumcision (data not presented). Such exposure to knowledge among the educated men could also explain the higher willingness to accept the intervention [33, 34].

The strengths of this study are that it is based on data from a nationally representative sample of uncircumcised men with high response rates. The socio-demographic characteristics of the weighted sample of men are similar to the national demographic profile and the results can thus probably be generalised to the adult male population in Uganda. This study uses PRRs in measuring associations, which are more conservative than the commonly used prevalence odds ratios in many studies of this kind [35, 36]. Although the observed risk ratios are not very high, they are more credible. However, there are some limitations. This is based on cross-sectional survey data and causal inferences cannot be drawn. It is also worth noting that expressed willingness may not necessarily lead to actual circumcision although behaviours often begin with intention. The study findings could also be limited by social desirability bias in men’s self-reporting of sexual risk behaviours in face to face interviews and recall bias when reporting on a 12 months period [27]. However, social desirability bias in underreporting sexual risk behaviours is more likely to affect women than men [29, 37] in the typical Ugandan context given that women are socially expected to have less adventurous sexual lifestyles [38]. The interviews were conducted by well-trained male interviewers, further reducing the risk of such bias. If the biases exist, they are likely to be non-differential because reporting of sexual behaviour was not likely in any way to be linked with reporting willingness to circumcise. The findings are also consistent with other studies in the sub Saharan African region [24, 25], further indicating their validity.

In conclusion, the findings from this study indicate higher likelihood of willingness to be circumcised among men with more sexual risk taking behaviours in Uganda. This indicates that the potential early adopters of male circumcision may be those in the greatest need of such an added protective measure. However, this does not imply that further promotion of SMC to reach the late adopters is not needed. Considering the high level of risk behaviour among potential early adopters, sustained efforts by the Ministry of Health and partners to sensitise and educate men undergoing circumcision on the importance of continued use of condoms are necessary to avoid risk compensation after the circumcision procedure.

## References

[1] UNAIDS, The Gap Report. 2014, Joint United Nations Programme on HIV/AIDS: Geneva.

[2] Wabwire-Mangen F., Odiit M., Kirungi W., Kaweesa K., D., and Wanyama O., J., Uganda HIV Prevention Response and Modes of Transmission Analysis. 2009, Uganda National AIDS Commission, UNAIDS: Kampala.

[3] Siegfried N., Muller M., Volmink J., Deeks J., Egger M., Low N., et al, Male circumcision for prevention of heterosexual acquisition of HIV in men. Cochrane Database Syst Rev, 2003(3): p. CD003362 12917962 10.1002/14651858.CD003362

[4] Auvert B., Buve A., Ferry B., Carael M., Morison L., Lagarde E., et al, Ecological and individual level analysis of risk factors for HIV infection in four urban populations in sub-Saharan Africa with different levels of HIV infection. AIDS, 2001 15 Suppl 4: p. S15–30. 11686462 10.1097/00002030-200108004-00003

[5] Weiss H.A., Quigley M.A., and Hayes R.J., Male circumcision and risk of HIV infection in sub-Saharan Africa: a systematic review and meta-analysis. AIDS, 2000 14(15): p. 2361–70. 11089625 10.1097/00002030-200010200-00018

[6] Gray R.H., Kigozi G., Serwadda D., Makumbi F., Watya S., Nalugoda F., et al, Male circumcision for HIV prevention in men in Rakai, Uganda: a randomised trial. Lancet, 2007 369(9562): p. 657–66. 17321311 10.1016/S0140-6736(07)60313-4

[7] Bailey R.C., Moses S., Parker C.B., Agot K., Maclean I., Krieger J.N., et al, Male circumcision for HIV prevention in young men in Kisumu, Kenya: a randomized controlled trial. Lancet, 2007 369(9562): p. 643–656. 17321310 10.1016/S0140-6736(07)60312-2

[8] Auvert B., Taljaard D., Lagarde E., Sobngwi-Tambekou J., Sitta R., and Puren A., Randomized, controlled intervention trial of male circumcision for reduction of HIV infection risk: the ANRS 1265 Trial. PLoS Med, 2005 2(11): p. e298 16231970 10.1371/journal.pmed.0020298 PMC1262556

[9] Auvert B., Sobngwi-Tambekou J., Cutler E., Nieuwoudt M., Lissouba P., Puren A., et al, Effect of male circumcision on the prevalence of high-risk human papillomavirus in young men: results of a randomized controlled trial conducted in Orange Farm, South Africa. J Infect Dis, 2009 199(1): p. 14–9. 10.1086/595566 19086814 PMC2821597

[10] Tobian A.A., Serwadda D., Quinn T.C., Kigozi G., Gravitt P.E., Laeyendecker O., et al, Male circumcision for the prevention of HSV-2 and HPV infections and syphilis. N Engl J Med, 2009 360(13): p. 1298–309. 10.1056/NEJMoa0802556 19321868 PMC2676895

[11] Wawer M.J., Tobian A.A., Kigozi G., Kong X., Gravitt P.E., Serwadda D., et al, Effect of circumcision of HIV-negative men on transmission of human papillomavirus to HIV-negative women: a randomised trial in Rakai, Uganda. Lancet, 2011 377(9761): p. 209–18. 10.1016/S0140-6736(10)61967-8 21216000 PMC3119044

[12] WHO/UNAIDS, New Data on Male Circumcision and HIV Prevention: Policy and Programme Implications. 2007, World Health Organisation and Joint United Nations Programme on HIV/AIDS,: Geneva.

[13] WHO/UNAIDS, Joint Strategic Action Framework to Accelerate the Scale-Up of Voluntary Medical Male Circumcision for HIV Prevention in Eastern and Southern Africa 2012–2016. 2011, World Health Organisation and Joint United Nations Programme on HIV/AIDS,.

[14] MOH, Safe male circumcision for HIV prevention National communication strategy. 2010, Ministry of Health: Kampala, Uganda.

[15] Uganda AIDS Commission, HIV and AIDS Uganda Country Progress Report 2013. 2014, UAC: Kampala, Uganda.

[16] Center for Communication Programs, The Health Communication Partnership Uganda, Final Report. 2012, Johns Hopkins Bloomberg School of Public Health, CCP: Kampala.

[17] Health Communication Partnerships, *HCP* 2010 Survey Highlights: Safe male circumcision for HIV prevention 2010, HCP: Kampala.

[18] MUSPH, School of Public Health, Makerere University College of Health Sciences Annual Report Aug 2009—July 2010. 2010, Makerere University: Kampala.

[19] MOH and ICF International, Uganda AIDS Indicator Survey 2011. 2012, Ministry of Health and ICF International: Kampala, Uganda and Calverton Maryland, USA.

[20] WHO UNAIDS and UNICEF, *Global HIV/AIDS Response: Epidemic update and health sector progress towards Universal Access* *Progress report*. 2011.

[21] WHO, Global update on the health sector response to HIV, 2014 2014, World Health Organization: Geneva, Switzerland

[22] Rogers E.M., Diffusion of Innovations. Third ed. 1983, New York: The Free Press.

[23] Westercamp N., Agot K., Jaoko W., and Bailey R.C., Risk compensation following male circumcision: results from a two-year prospective cohort study of recently circumcised and uncircumcised men in Nyanza province, Kenya. AIDS Behav, 2014 18(9): p. 1764–75. 10.1007/s10461-014-0846-4 25047688

[24] Chikutsa A., Ncube C.A., and Mutsau S., Association between wanting circumcision and risky sexual behaviour in Zimbabwe: evidence from the 2010–11 Zimbabwe demographic and health survey. Reproductive Health, 2015 12(15). 10.1186/s12978-015-0001-3 PMC4364469 25889318

[25] Keetile M. and Rakgoasi S.D., Male Circumcision; willingness to undergo safe male circumcision and HIV risk behaviors among men in Botswana. African Population Studies, 2014 28(3): p. 1345–61.

[26] UNAIDS, National AIDS Programmes: A Guide to Monitoring and Evaluation. 2000, Joint United Nations Programme on HIV/AIDS: Geneva, Switzerland.

[27] Kelly C.A., Soler-Hampejsek E., Mensch B.S., and Hewett P.C., Social desirability bias in sexual behavior reporting: evidence from an interview mode experiment in rural Malawi. Int Perspect Sex Reprod Health, 2013 39(1): p. 14–21. 10.1363/3901413 23584464 PMC4023461

[28] Mnyika K.S., Klepp K.I., Kvale G., and Ole-Kingori N., Determinants of high-risk sexual behaviour and condom use among adults in the Arusha region, Tanzania. Int J STD AIDS, 1997 8(3): p. 176–83. 9089028 10.1258/0956462971919840

[29] Schroder K.E.E., Carey M.P., and Vanable P.A., Methodological Challenges in Research on Sexual Risk Behavior: II. Accuracy of Self-Reports. Annals of Behavioral Medicine: A Publication of the Society of Behavioral Medicine, 2003 26(2): p. 104–123. 10.1207/s15324796abm2602_03 PMC2441938 14534028

[30] Cassell M.M., Halperin D.T., Shelton J.D., and Stanton D., Risk compensation: the Achilles' heel of innovations in HIV prevention? BMJ, 2006 332(7541): p. 605–7. 16528088 10.1136/bmj.332.7541.605 PMC1397752

[31] UBOS and ICF International, Uganda Demographic and Health Survey 2011. 2012, Uganda Bureau of Statistics (UBOS) and ICF International Inc.: Kampala, Uganda and Calverton, Maryland.

[32] Kitara D., Lagoro, Ocero A., Lanyero J., and Ocom F., Roll-out of Medical Male circumcision (MMC) for HIV prevention in non-circumcising communities of Northern Uganda. The Pan African Medical Journal, 2013 15(100). 10.11604/pamj.2013.15.100.2338 PMC3810160 24198894

[33] Westercamp M., Agot K.E., Ndinya-Achola J., and Bailey R.C., Circumcision preference among women and uncircumcised men prior to scale-up of male circumcision for HIV prevention in Kisumu, Kenya. AIDS Care, 2012 24(2): p. 157–66. 10.1080/09540121.2011.597944 21854351 PMC3682798

[34] Muhangi D., Factors that Influence Decisions to Seek Medical Male Circumcision Services: A Report of Qualitative Research in Kampala, Kayunga, Pallisa, Kasese and Mbale Districts—Uganda. 2010, USAID/JHU: Kampala, Uganda.

[35] Barros A.J. and Hirakata V.N., Alternatives for logistic regression in cross-sectional studies: an empirical comparison of models that directly estimate the prevalence ratio. BMC Med Res Methodol, 2003 3: p. 21 14567763 10.1186/1471-2288-3-21 PMC521200

[36] Axelson O., Fredriksson M., and Ekberg K., Use of the prevalence ratio v the prevalence odds ratio as a measure of risk in cross sectional studies. Occup Environ Med, 1994 51(8): p. 574 7951785 10.1136/oem.51.8.574 PMC1128040

[37] Kelly C.A., Hewett P.C., Mensch B.S., Rankin J.C., Nsobya S.L., Kalibala S., et al, Using biomarkers to assess the validity of sexual behavior reporting across interview modes among young women in Kampala, Uganda. Stud Fam Plann, 2014 45(1): p. 43–58. 10.1111/j.1728-4465.2014.00375.x 24615574 PMC4528964

[38] Brawley E.M., The Relationship Between Gender Norms and Expectations and the Sexual Practices of Ugandan Men, in Health Services. 2006, University of Washington: Washington.

