# Correction: Sexual Risk Behaviours and Willingness to Be Circumcised among Uncircumcised Adult Men in Uganda

**DOI:** 10.1371/journal.pone.0146507

**Published:** 2016-01-04

**Authors:** Simon P. S. Kibira, Fredrick Makumbi, Marguerite Daniel, Lynn Muhimbuura Atuyambe, Ingvild Fossgard Sandøy

The reference cited in the fourth sentence of the “Statistical analyses” section are incorrect. The sentence should read: PRRs were used because the outcome variable had prevalence above 10% (Zocchetti et al., 1997; Traissac et al., 1999; Barros et al., 2003).

The correct references are:

Zocchetti, C., D. Consonni, and P.A. Bertazzi, *Relationship between prevalence rate ratios and odds ratios in cross-sectional studies*. Int J Epidemiol, 1997. **26**(1): p. 220–3.

Traissac, P., Y. Martin-Prevel, F. Delpeuch, and B. Maire, *[Logistic regression vs other generalized linear models to estimate prevalence rate ratios]*. Rev Epidemiol Sante Publique, 1999. **47**(6): p. 593–604.

Barros, A.J. and V.N. Hirakata, *Alternatives for logistic regression in cross-sectional studies*: *an empirical comparison of models that directly estimate the prevalence ratio*. BMC Med Res Methodol, 2003. **3**: p. 21.
